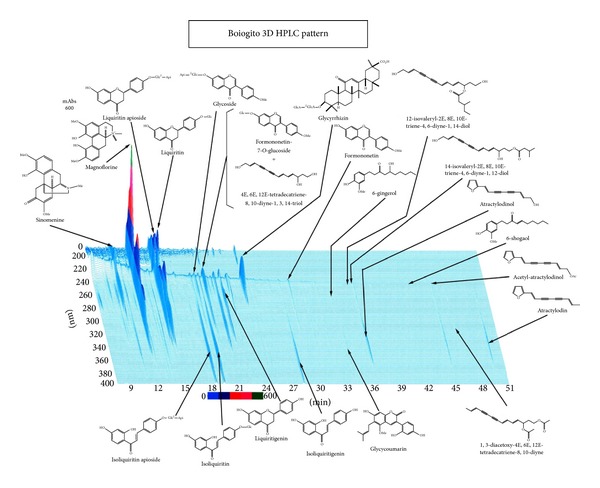# Significance of Kampo, Japanese Traditional Medicine, in the Treatment of Obesity: Basic and Clinical Evidence

**DOI:** 10.1155/2013/943075

**Published:** 2013-04-15

**Authors:** Jun-ichi Yamakawa, Junji Moriya, Kenji Takeuchi, Mio Nakatou, Yoshiharu Motoo, Junji Kobayashi

**Affiliations:** ^1^Department of General Medicine, Kanazawa Medical University, 1-1 Daigaku, Uchinada, Kahoku District Ishikawa 920-0293, Japan; ^2^Department of Anesthesia, Fukuiken Saiseikai Hospital, Fukui, Japan; ^3^Department of Medical Oncology, Kanazawa Medical University, Ishikawa, Japan

## Abstract

The cause of obesity includes genetic and environmental factors, including cytokines derived from adipocytes (adipo-cytokines). Although drug therapy is available for obesity, it is highly risky. Our main focus in this review is on the traditional form of Japanese medicine, Kampo, in the treated of obesity. Two Kampo formulas, that is, bofutsushosan (*防風通聖散*) and boiogito (*防己黄耆湯*), are covered by the national health insurance in Japan for the treatment of obesity. Various issues related to their action mechanisms remain unsolved. Considering these, we described the results of basic experiments and presented clinical evidence and case reports on osteoarthritis as examples of clinical application of their two Kampo medicine. Traditional medicine is used not only for treatment but also for prevention. In clinical practice, it is of great importance to prove the efficacy of combinations of traditional medicine and Western medicine and the utility of traditional medicine in the attenuation of adverse effects of Western medicine.

## 1. Background

### 1.1. Historical Background

Until the 20th century, Japanese herbal medicine (Kampo medicine) had not been recognized as a useful tool for the treatment of obesity. Since the latter half of the 20th century, great attention has been paid to upper-body obesity or visceral obesity as a risk factor for several lifestyle-related diseases. In the 1980s and 1990s, several investigators proposed that dyslipidemia, high blood glucose, and high blood pressure were important risks for cardiovascular diseases due to metabolic disorders [1–3].

These days, this combination of disorders (i.e., dyslipidemia, hypertension, and hyperglycemia) is indicative of “metabolic syndrome.” The initial cause of the metabolic syndrome or abdominal obesity is likely to be visceral fat accumulation (which is regulated by both genetic and environmental factors). It is generally recognized that the accumulation of visceral fat causes the secretion of adiponectin to decrease and the so-called bad adipocytokines (plasminogen activator inhibitor-1 [PAI-1] [4–6], tumour necrosis factor-*α* [TNF-*α*] [7–9], angiotensinogen, etc.) to increase. Proper diet and exercise are the basic therapeutic approaches to reduce abdominal obesity. Medications are considered afterwards.

### 1.2. Antiobesity Medication

Only one antiobesity medication orlistat (Xenical) is currently approved by the Food and Drug Administration (FDA) for long-term use [10–15]. It reduces intestinal fat absorption by inhibiting pancreatic lipase. Rimonabant (Acomplia), a second drug, works via specific blockade of the endocannabinoid system. Its development is originated from the knowledge that cannabis smoking often increases hunger, which is often referred to as “the munchies”. Its use in the treatment of obesity had been approved in Europe but not in the USA or Canada due to safety concerns [16–20]. In October 2008, the European Medicines Agency recommended the suspension of the sale of rimonabant as the risks seemed to be greater than the benefits [21]. In October 2010, sibutramine (Meridia), which acts in the brain to inhibit neurotransmitters deactivation and thereby appetite, was withdrawn from the USA and Canadian markets due to cardiovascular concerns [11, 22]. Because of their potential side effects, anti-obesity drugs should only be prescribed for obesity when the benefits outweigh the risks of treatment [23, 24].

Hasani-Ranjbar et al. [25] have reported a systematic review of the efficacy and safety of herbal medicines used in the treatment of obesity. This review focuses on the efficacy and safety of effective herbal medicines in the management of obesity in humans and animals. Of the publications identified in the initial database, 915 results were identified and reviewed, and a total of 77 studies were included (19 human and 58 animal studies). Studies with *Cissus quadrangularis, Sambucus nigra, Asparagus officinalis, Garcinia atroviridis*, and ephedra and caffeine, Slimax (extract of several plants including *Zingiber officinale* and bofutsushosan) showed a significant decrease in body weight. In 41 animal studies, significant weight loss or inhibition of weight gain was found. No significant adverse effects or mortality were observed except in studies with supplements containing ephedra, caffeine, and bofutsushosan.

Given this background, our focus will be on Kampo as the treatment for obesity [26, 27].

## 2. The Status of Kampo Medicine in the Treatment of Obesity

### 2.1. The Definition of Obesity

 In 1997, the World Health Organization (WHO), in cooperation with the International Obesity Task Force (IOTF), defined normal weight as body mass index (BMI) from 18.5 to <25 kg/m^2^ and obesity as BMI ≥25 kg/m^2^. According to the Japan Society for the Study of Obesity criteria, obesity disease is diagnosed when individuals have obesity-associated disease or visceral fat area on abdominal CT scan equal to or greater than 100 cm^2^ [28]. 

### 2.2. Kampo Medicine Provided under Health Insurance in Japan

 The Kampo medicine of “traditional medical treatment” in Japan is in special environment. In a covered-medical-services system of Japan, getting a medical license for per the Forney Western medicine is mandate to prescribe Kampo medicine. Medical treatment is performed with the same stance as Western medicine. However, it is very difficult to understand Kampo medicine. Terasawa has indicated the outline of Kampo medicine to the first issue of this journal [29–31]. The universal healthcare system was established in Japan in 1961, and 4 Kampo extracts were approved as prescribable medications in 1967. That number is now 148. The application increased, and according to a survey by the Journal Nikkei Medical, more than 70% of physicians prescribe Kampo drugs today [32]. Physicians in Japan are entitled to prescribe both Western and Kampo medicines. Among the 148 Kampo formulas, only 2 are for the treatment of obesity. The concept of obesity is not described in classical Kampo. However, eating too much sweet food has been described as the cause of diabetes. On the other hand, how to manage and treat lifestyle-related disease is well described in classical Kampo medicine. In this paper, we focus on the two Kampo formulations used in the treatment of obesity, bofutsushosan (*防風通聖散*) and boiogito (*防己黄耆湯*).

### 2.3. Bofutsushosan

**Figure 1:**
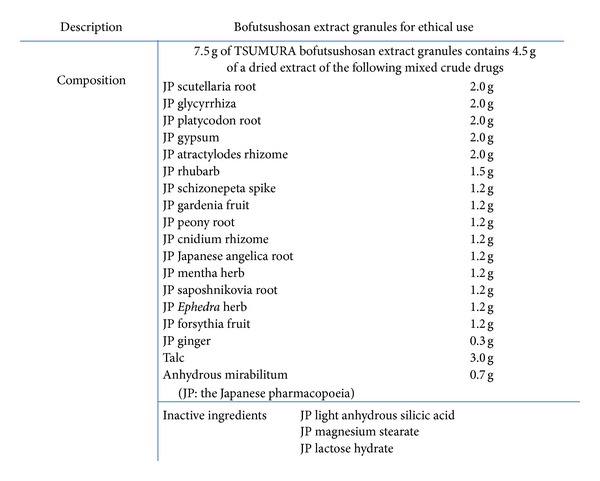


**Figure 2:**
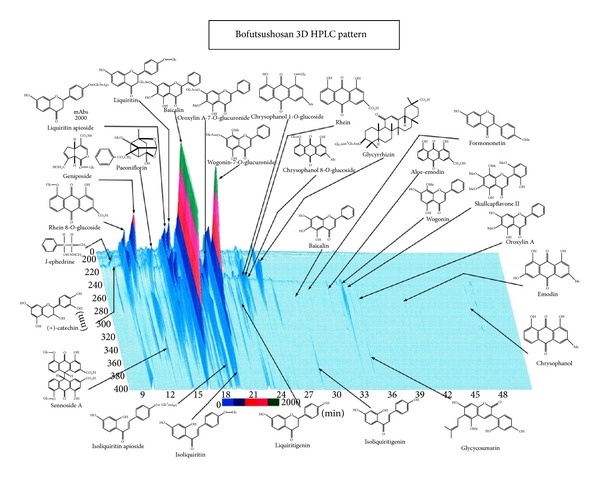


Chemical composition and HPLC fingerprint are shown in Figures 1 and 2. Bofutsushosan is indicated for the relief of the following symptoms in patients with thick subcutaneous abdominal fat and a tendency toward constipation: hypertension (palpitation, shoulder stiffness, and hot flushes), obesity, swelling, and constipation. The usual adult dose is 7.5 g/day orally in 2 or 3 divided doses before or between meals. The dosage may be adjusted according to the patient's age, body weight, and symptoms. Bofutsushosan should be administered with care in patients with the following conditions: (1) diarrhea or soft feces (these symptoms may be aggravated), (2) a weak gastrointestinal tract (anorexia, epigastric distress, nausea, vomiting, abdominal pain, soft feces, and diarrhea may occur), (3) anorexia, nausea, or vomiting (these symptoms may be aggravated), (4) a period of weakness after disease or with a greatly weakened constitution (adverse reactions are likely to occur, and the symptoms may be aggravated by treatment), (5) a marked tendency to sweat (excess sweating and/or generalized weakness may occur), (6) cardiovascular disorders including angina pectoris and myocardial infarction or those with a history of such disorders, (7) severe hypertension, (8) severe renal dysfunction, (9) dysuria, and (10) hyperthyroidism. The diseases and symptoms mentioned in (6)–(10) may be aggravated by treatment. The source is *Manbyo-kaishun-Chufumon *(*万病回春*, *中風門*). The prescription was used in Ikkando (*一貫堂*) medicine, which was systematized by Dohaku Mori (1867–1931). Bofutsushosan is standard for apoplectic patients, whose appearance suggests they are at risk of future cerebral hemorrhage, and are slightly obese, have a paunch, and have a sturdy build with pale yellow skin color. Bofutsushosan is used for people with solid build, slight obesity, thick abdominal subcutaneous fat, strong intestines, and good appetite. They are more prone to constipation who suffer inflammation in the nose and throat because the whole body is susceptible to heating up, and they tend to have high blood pressure with sensitivity to heat.

### 2.4. Boiogito

**Figure 3:**
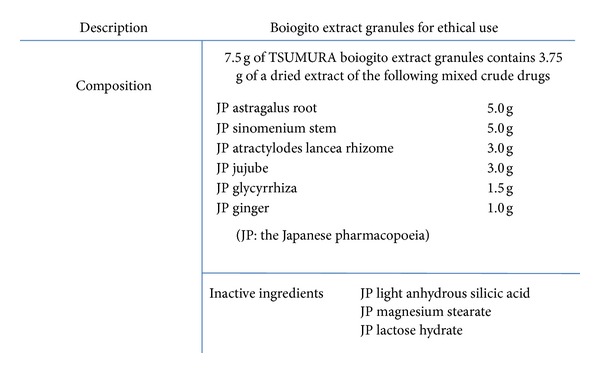


 Chemical composition and HPLC fingerprint are shown in Figures 3 and 4. Boiogito is indicated for the relief of the following symptoms in white-complexioned, soft-muscled, flabby patients who are easily fatigued, perspire profusely, do not excrete enough urine, and develop edema in the lower limbs, knee joint swelling, and pain: nephritis, nephrosis, nephropathy of pregnancy, hydrocele testis, obesity, arthritis, carbuncles, furuncles, myositis, edema, dermatosis, hyperhidrosis, and menstrual irregularity. The usual adult dose is 7.5 g/day orally in 2 or 3 divided doses before or between meals. The dosage may be adjusted according to the patient's age and body weight and symptoms. (1) When this product is used, the patient's “*Sho*” (*証*, patterns) should be taken into account. The patient's progress should be carefully monitored, and if symptoms/findings do not improve, continuous treatment should be avoided. (2) Since this product contains glycyrrhiza (*甘草*, kanzo), careful attention should be paid to the serum potassium level, blood pressure, and so forth, and if any abnormality is observed, administration should be discontinued. (3) When this product is coadministered with other Kampo preparations (Japanese traditional herbal medicines) and so forth, attention should be paid to duplication of crude drug contents. Sho: the term “Sho” refers to a particular pathological status (pattern of symptoms) determined by Kampo diagnosis. The pattern is based on the patient's constitution, symptoms, and so forth. Kampo preparations (Japanese traditional herbal medicines) should be used after their suitability for “Sho” has been confirmed. The source is the *Kinkiyoryaku *(*金匱要略*): convulsion, dampness, and heatstroke diseases—water qi diseases (*痙湿暍病篇*·*水氣病編*). It says “boiogito is chiefly used for people suffering neuralgia, floating pulse, heavy body, and sweating with aversion to wind. The external signs for boiogito include wind edema and floating pulse. The patient may appear to have a sweaty head, but no other external signs, except the lower body, feels heavy with edema extending to the groin, making bending and extending difficult, and yet suffer no ill effects above the low back.” In other words, it is used for patients with the so-called flabby constitution, pale complexion, proneness to fatigue, sweatiness, and decreased urine output. Boiogito is effective for patients who often suffer arthralgia or low back pain, who are susceptible to edema and sensitive to cold, who demonstrate the so-called “frog belly” when lying down, and who have abdominal skin that shows dimpling, softness (deficiency *Nankyo*) when pinched, and flabbiness. Boiogito is more often used in women than men. Patients are pale, plump, flabby, and heavy; their demeanor is listless; they shy away from cleaning and cooking, move infrequently, and eat little. Patients generally pass stools daily, have low menstrual flow, and may complain of irregular menses. They readily perspire, and in summer, their perspiration is profuse. Edema develops in the legs to the degree that shoes and socks are tight by the end of the day.

### 2.5. Other Prescriptions

Daisaikoto (*大柴胡湯*) is used for obese patients with muscular build and robust appetite owing to their active lifestyle. When faced with stress or unpleasantness, patients readily develop liver qi depression, irritability, irascibility, a bitter taste in the mouth, and blood congestion in the eye. Transverse invasion of liver qi into the stomach upsets splenogastric function and abnormally promotes appetite, which in turn adds to the obesity [30]. Tokakujokito (*桃核承氣湯*) and keishibukuryogan (*桂枝茯苓丸*) are commonly used for women with sudden weight gain in menopause. In patients with blood stasis (Oketsu), the blood rises to the face turning the face and mucous membranes of the tongue and lips red. Venous engorgement and telangiectasia of the skin and mucous membranes is associated with dry, rough skin, lower abdominal bloating, resistance, and tenderness and autonomic symptoms such as upper heat and lower cold (*Hienobose*). Patients also often complain of numbness and pain in various parts of the body.

## 3. Basic Research of Kampo

### 3.1. Introduction of Basic Research

Numerous investigators are attempting to clarify how Kampo exerts its effects. However, many issues need to be resolved before the mechanisms are clearly understood. First, as shown by the data on HPLC, many of the components of Kampo have effects. Also, HPLC analysis does not indicate volatile-element composition. Components may chemically react when mixed. Thus, it is hard to show which of the components in Kampo the active components are. Second, Kampo are components considered to be prodrugs, which exert their effects after being metabolized by the human body. On the other hand, some exert their effects immediately after oral administration.

### 3.2. Bofutsushosan

*Ephedra* herb (*麻黄*, mao) is one of the most important natural remedies in Kampo. Ephedrine, a main component of *Ephedra* herb, activates adrenalin receptors in sympathetic nerves, leading to increased production of cAMP and in turn to increased heat production from brown adipose tissue [33–35]. Nakayama et al. have reported bofutsushosan that seems effective in the activities of antiobesity, antihyperlipidemia, and antihyperlipids in liver cytoplasm [33]. In France, ephedrine combined with caffeine has been used for the treatment of obesity, and the action mechanism is considered to be phosphodiesterase inhibition [36]. Yoshida et al. have reported the antiobesity action of bofutsushosan in monosodium glutamate (MSG) obese mice [34]. The aim was to investigate whether the antiobesity action of bofutsushosan is due to the stimulation of brown adipose tissue thermogenesis and inhibition of phosphodiesterase activity. Bofutsushosan works by stimulating BAT thermogenesis and inhibiting phosphodiesterase activity in mice. Akagiri et al. have reported that bofutsushosan, an oriental herbal medicine, attenuates the weight gain of white adipose tissue and the increased size of adipocytes associated with the increase in their expression of uncoupling protein 1 in high-fat diet-fed male KK/Ta mice. Bofutsushosan decreases the weight and size gains of WAT along with upregulating UCP1 mRNA in WAT in high-fat diet-fed mice [37]. Shimada et al. have reported preventive effects of bofutsushosan on obesity and various metabolic disorders. In the TSOD mice treated with bofutsushosan, body weight gain and visceral/subcutaneous fat accumulation were significantly suppressed. Biochemical parameters in plasma (glucose, TC, insulin, and tumor necrosis factor-alpha level) were significantly suppressed, and abnormal glucose tolerance, elevation of blood pressure, and peripheral neuropathy accompanying progression of metabolic disorders were also significantly suppressed [27].

### 3.3. Boiogito

 There are few basic experimental studies on boiogito. Shimada et al. have reported preventive effect of boiogito on metabolic disorders in the TSOD mouse, a model of spontaneous obese type II diabetes mellitus. boiogito is effective as an antiobesity drug for the “asthenic constitution” type in which subcutaneous fat accumulates but cannot be expected to exert a preventive effect against various symptoms of metabolic syndrome that are based on visceral fat accumulation [26]. We previously reported that boiogito had antiobesity action in ovariectomized rats [38]. In this experiment, the antiobesity properties of boiogito were evaluated in ovariectomized rats by measuring changes in levels of serum cytokines and fat cell adipocytokines. After treatment with boiogito for 6 weeks (20-week-old rats), there was a significant weight decrease compared to the control group, a significant dose-dependent increase in serum tumor-necrosis-factor- (TNF-)*α* level, and a significant increase in adipose-tissue TNF-*α* level, suggesting that boiogito contributes to weight gain inhibition via the secretion of TNF-*α* by fat cells. On the other hand, peroxisome proliferator-activated receptor-*γ* and adiponectin protein levels did not differ significantly between experimental and control groups; the levels of their corresponding mRNAs tended to increase dose dependently, and the level of resistin did not change significantly.

Our previous study using rat preadipocytes suggests that bofutsushosan and boiogito inhibit differentiation and proliferation of white adipocytes through distinct mechanisms [39]. Three Kampo medicines, boiogito, bofutsushosan, and orengedokuto used for treatment of obesity were investigated to determine their effects on adipogenesis in cultured rat white adipocytes. Administration of the three extracts (1–100 mg/mL) suppressed adipogenesis in a concentration-dependent manner without any cytotoxicity. The three herbal extracts were found to have the potential to prevent adipogenesis in rat white adipocytes. Different mechanisms modulating gene expression levels were involved.

## 4. Clinical Application of Kampo

### 4.1. Introduction of Evidence-Based Medicine (EBM)

 Two randomized controlled trials (RCTs) of bofutsushosan show its potential as an antioxidant. Ogawa et al. conducted a double-blind (DB) RCT on the effect of bofutsushosan on the lag time of low-density lipoprotein (LDL) oxidation in healthy individuals [40]. Antioxidants are present in herbs or crude herbal formulations. The effect of bofutsushosan on *ex vivo* LDL oxidation lag time was studied in healthy human subjects. Although bofutsushosan had no detectable systemic antioxidative effects, *ex vivo* results suggested its antioxidative effect on LDL oxidation. The RCT by Hioki et al. demonstrated the effect and safety of bofutsushosan in Japanese obese subjects [41]. The aim was to determine whether bofutsushosan could decrease visceral adiposity and insulin resistance. They concluded that bofutsuhosan could be a useful herbal medicine in treating obesity with impaired glucose tolerance.

### 4.2. Case of Knee Osteoarthritis

After receiving sufficient medical treatment from an orthopedist, and also in order to raise a patient's quality of life (QOL), they introduce to a Kampo medicine medical specialist. Knee osteoarthritis is a degenerative disease of the knee joint that is more common in people older than 40 years and in women. The most important characteristic of knee osteoarthritis is the degeneration of the knee joint articular cartilage, causing decreased QOL in affected people. Obesity is one of the most important causes of osteoarthritis. We report the two cases of obese patients who showed marked improvement in osteoarthritis-related clinical symptoms as a result of Kampo treatment.

**Case 1 (a 64-year-old female)**

Chief complaints: bilateral articular pain in the knee. Past history: hypertension, hyperlipidemia, obesity, and osteoarthritis. Present illness: from the age of 42 year, she underwent dietary therapy for obesity, which was not remarkably effective. She was referred to our department for Kampo therapy on May 12, 2004. Physical findings: body height 143 cm, body weight 76.4 kg, and BMI 37.4 kg/m^2^. Oriental medical diagnosis: interior heat excess pattern (eight principles classification), sunken excessive pulse (pulse diagnosis), yellow slimy tongue fur (tongue diagnosis), and excessive abdominal strength, slight gastric stuffiness, and paunch (abdominal examination); therefore, bofutsushosan (TJ-62) 7.5 g t.i.d. was prescribed (pattern-based diagnosis). Clinical course: body weight was decreased by 4 kg (from 76 kg to 72 kg) in 14 days after the start of bofutsushosan (TJ-62) 7.5 g t.i.d. There was a remarkable improvement in leg edema and bowel movement. On day 28, her body weight was 70 kg, and she no longer needed a painkiller prescribed by her orthopedist for knee osteoarthritis.

**Case 2 (a 76-year-old female)**

Chief complaints: poor condition of the knee. Past history: hyperlipidemia and osteoarthritis. Present illness: she had been treated for hyperlipidemia by her family doctor for a long time. four years ago, she received a diagnosis of knee osteoarthritis and was treated accordingly. She visited our department to receive Kampo treatment on April 20, 2007. Physical findings: body height 151 cm, body weight 60.3 kg, and BMI 26.4 kg/m^2^. Oriental medical diagnosis: eight network classification: imaginary cold proof back. Pulse diagnosis: slightly floating. Tongue diagnosis: wet, frank color, and thin white moss. Abdominal examination: belly force: imaginary frog belly leg edema (+) and Based on sui testimony diagnosis, the treatment with boiogito (TJ-20) 7.5 g t.i.d. was initiated. Clinical course: two weeks after the administration of boiogito, knee joint pain was improved, and body weight reduced 2 kg (from 60 to 58 kg). One month after administration, she no longer needed the painkiller prescribed by her orthopedist.

In Case 1, the diagnosis was the hyperfunctioning type of febrile syndrome of the viscera (according to the four paired parameters of Kampo diagnosis) and the so-called “muscular type,” which is associated with constipation and dizziness. Bofutsushosan was prescribed because of her marked obesity (BMI 37.4), and it proved to be very effective. In Case 2, the diagnosis was the hypofunctioning type of febrile syndrome of the viscera (according to the four paired parameters of Kampo diagnosis) and the so-called “white complexion and flabby body type.” She had edema and excessive sweating with weak stomach. We prescribed boiogito. Without body movement, no energy is consumed. The lack of exercise reduces the amount of muscle producing energy and, in turn, basal metabolism leading to resistance of the body to energy consumption. Notably, the lack of exercise impacts the reduction of basal metabolism more than it does with the reduction of energy consumption. It is generally accepted that environmental factors as well as genetic factors influence the development of obesity. Still, it is not easy to manage obesity. Lifestyle modifications, such as dietary and exercise interventions, are hard to follow. For obese subjects with knee problems, walking exercise is practically impossible. Thus, any suggestion by family members or others that exercise is needed could impose a mental burden. Based on the findings in the above-mentioned two cases, we suggest that the herbal treatment may trigger the awareness of weight loss. Majima et al. have reported the effect of the Japanese herbal medicine, Boiogito, on the osteoarthritis of the knee with joint effusion. boiogito have a possibility for a treatment modality for joint effusion with osteoarthritis of the knee [42].

## 5. Conclusion

Modern Western medicine is the official medicine practiced in every country. In Kampo medicine as well as other traditional medicines, different formulas have been prescribed for patients with the same disease, and diagnosis has been made by considering the constitution and condition of each patient. Such an individualized treatment has been successful in patients without any particular abnormalities of laboratory data. WHO is rigorously trying to incorporate complementary medicine into conventional medicine, emphasizing the importance of traditional medicine. Kampo is expected to be applied not only to therapeutics but also to disease prevention. In clinical practice, the usefulness of Kampo in combination with Western medicine is to be confirmed.

## Figures and Tables

**Figure 4:**